# Complexity in radiological morphology predicts worse prognosis and is associated with an increase in proteasome component levels in clear cell renal cell carcinoma

**DOI:** 10.3389/fonc.2022.1039383

**Published:** 2022-12-08

**Authors:** Kohei Kobatake, Kenichiro Ikeda, Jun Teishima, Yohei Sekino, Takashi Babasaki, Yuki Kohada, Ryo Tasaka, Kenshiro Takemoto, Takafumi Fukushima, Shunsuke Miyamoto, Hiroyuki Kitano, Keisuke Goto, Keisuke Hieda, Tetsutaro Hayashi, Nobuyuki Hinata

**Affiliations:** ^1^ Department of Urology, Graduate School of Biomedical and Health Sciences, Hiroshima University, Hiroshima, Japan; ^2^ Department of Urology, Kobe City Hospital Organization Kobe City Medical Center West Hospital, Kobe, Japan

**Keywords:** proteosome, PSMB1, PSMB3, radiological morphology, renal cell carcinoma (RCC), clear cell renal cell carcinoma

## Abstract

**Background:**

We previously reported preoperative radiological morphology (RM) as an independent predictor for pathological upstaging after partial nephrectomy in patients with T1 renal cell carcinoma (RCC).

**Purpose:**

To investigate the prognostic importance of RM in all stages and the molecular characteristics underlying the differences between each type of RM in patients with clear cell RCC (ccRCC).

**Design, setting, and participants:**

The Cancer Imaging Archive datasets (TCIA), comprising CT images and RNA-sequencing data, were used (n = 163). Specimens from 63 patients with ccRCC at our institution and their CT images were used. All images were divided into three types according to RM classification.

**Outcome measurements and statistical analysis:**

Relationships with outcome were analyzed using Cox regression analysis and log-rank test.

**Results and limitations:**

The irregular type was a significant independent predictor of worse disease-free survival (odds ratio: 2.22, p = 0.037) compared to round and lobular types in TCIA datasets. The irregular type showed a significant increase in both mRNA and protein expression of proteasome components, PSMB1 and PSMB3. Moreover, high expression of their coding genes shortened the progression-free survival of the patients with ccRCC who received sunitinib or avelumab plus axitinib therapy. The study limitations include the qualitative classification of RM and the need for novel radiomics and texture analysis techniques.

**Conclusions:**

Investigating RM on pre-treatment CT scans can effectively predict worse prognosis. Increased RM complexity may indirectly predict drug sensitivity *via* increased expression of PSMB1 and PSMB3 in patients with ccRCC. Specific targeting of the ubiquitin-proteasome system might be a novel treatment strategy for ccRCC with increased RM complexity.

**Patient summary:**

The clinical and morphological characteristics of patients with ccRCC vary greatly according to cancer staging. In this study, we built upon our prior findings of the prognostic importance of RM in T1 RCC and expanded it to encompass all stages of RCC, using a series of patients from a Japanese hospital.

## Introduction

Renal cell carcinoma (RCC) is a chemotherapy-resistant malignancy distinguished by its histopathological features and underlying gene mutations (1). Clear cell RCC (ccRCC) is the most common RCC subtype, and most cases are characterized by the loss of chromosome 3p on which the von Hippel−Lindau gene lies (2). Management options for all RCC stages have exponentially increased over the past two decades (3); however, surgical resection remains the only curative treatment for localized RCC (4). Therefore, various preoperative prognostic models have been developed for radical or partial nephrectomy (3, 5).

Computed tomography (CT) is the first-line imaging modality used for staging locoregional and suspected metastatic disease, and radiomic analysis using contrast-enhanced CT has been widely performed (6). Contrast-enhanced CT examination allows for the detection of the distinctive quantifiable features that characterize RCC, including peak tumor enhancement, tumor heterogeneity, and percent contrast washout (7).

We previously divided the radiological morphology (RM) of clinical T1 RCC that required partial nephrectomy into three types based on their complexity on preoperative CT scans: round, lobular, and irregular (8). RM classification proved to be an independent predictive factor of pathological upstaging in patients with clinical T1 RCC since the irregular type had a significantly higher potential for pathological upstaging and a lower survival rate than other types.

We hypothesized that RM taxonomy, although qualitative, may be a prognostic factor not only for cT1 tumors but for all RCC stages. In this study, we examined the clinical and molecular characteristics of RM on preoperative CT scans in patients with different stages of ccRCC.

## Materials and methods

### Human data collection and processing

CT images and their associated datasets of The Cancer Genome Atlas - Kidney Renal Clear Cell Carcinoma (TCIA-KIRC) were downloaded from The Cancer Imaging Archive (TCIA, https://www.cancerimagingarchive.net/), which consists of the CT scans of 210 patients who underwent either partial or radical nephrectomy (9), and cBioPortal (https://www.cbioportal.org/). Of them, 163 ccRCCs were classified into the three categories (round, lobular, or irregular type) according to RM classification based on contrast-enhanced CT or plane CT images as previously described (8). Clinical information and genomic profiles were documented, and Gene Set Enrichment Analysis was used to compare gene expression profiles of all three ccRCC types. Kyoto Encyclopedia of Genes and Genomes (KEGG) gene sets were also used to compare RM types, and differences with *p <*0.05 were considered statistically significant. Clinical and genomic data from JAVELIN RENAL 101 have been provided by Motzer et al. (10). The 63 samples used for immunohistochemistry were collected from patients at Hiroshima University Hospital. Written comprehensive consent forms for basic and clinical research were obtained from each patient. This study was conducted in accordance with the Ethical Guidance for Human Genome/Gene Research of the Japanese Government. The Institutional Review Board of Hiroshima University Hospital approved this study (approval no. E-1800 and E-2065).

### Immunohistochemistry

A total of 63 tissue samples and the complete clinical data of patients with ccRCC who underwent partial or total (radical and cytoreductive) nephrectomy at Hiroshima University Hospital from 2006 to 2018 were collected for immunohistochemistry (IHC). In this cohort, six patients underwent partial nephrectomy while fifty-seven underwent radical or cytoreductive nephrectomy. All samples were formalin-fixed and paraffin-embedded. Samples were stained with human anti-PSBM1 (HPA029635, Sigma Aldrich) and human anti-PSMB3 (HPA042775, Sigma Aldrich) antibodies according to IHC data for PSMB1 and PSMB3 in The Human Protein Atlas (https://www.proteinatlas.org/). As normal cells in tubules have been found to be stained by both antibodies, we confirmed that tubules within normal tissue were stained in all cases and recorded as negative when no staining occurred or when only the cancer cell membrane was stained. Samples were recorded as positive when cytoplasmic staining or convincing intensity in the minority or majority of cancer cells was observed. Typical examples of staining of both normal tubules and cancer cells are shown in **Supplementary Figure 1**.

### Statistical analysis

Univariate and multivariate Cox regression analyses, unpaired *t*-test, χ^2^ test, and log-rank test were used to compare the groups. Multiple group comparisons were performed using a one-way analysis of variance. Log-rank test was used to compare survival. Statistical analyses were performed using GraphPad Prism 8 (GraphPad Software Inc., RRID: SCR_002798). Univariate and multivariate Cox regression analyses were performed using JMP (SAS Institute Inc., RRID: SCR_008567).

## Results

### Clinicopathological characteristics of each RM

**Figure 1:**
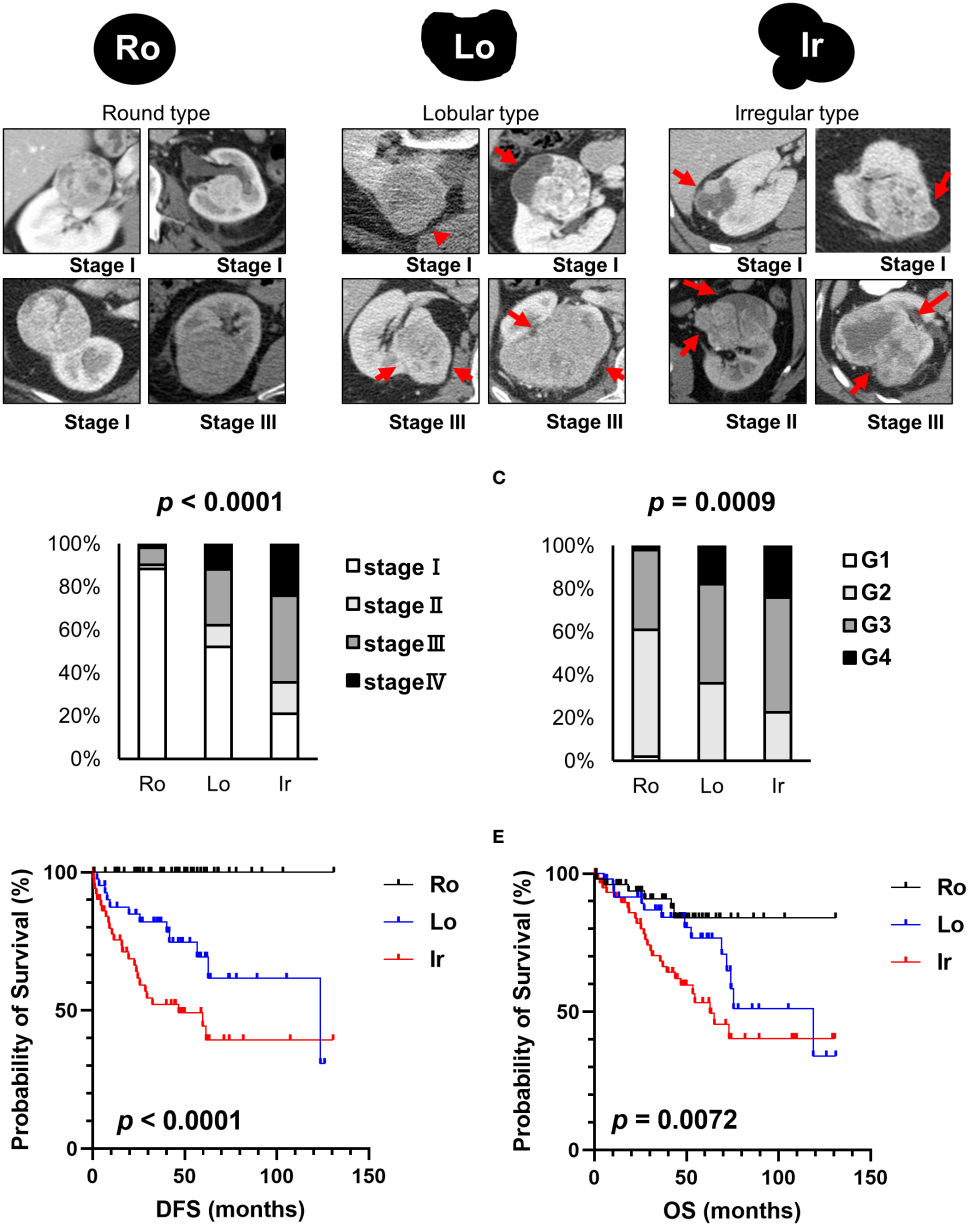
Clinical characteristics of each RM in TCIA datasets. **(A)** Schematic images and representative CT scans of the round, lobular, and irregular types from TCIA datasets. **(B)** The relationship between each RM and stage. **(C)** The relationship between each RM and grade. **(D)** Comparison of disease-free survival (DFS) between each RM type. **(E)** Comparison of overall survival (OS) between each RM type. Ro, round type; Lo, lobular type; Ir, irregular type.

Representative CT scan findings of 163 patients in TCGA-KIRC datasets from TCIA of round, lobular, and irregular ccRCCs are shown in **Figure 1A**. The clinicopathological features of the three types of RM were compared (**Table 1**). The number and the median age of patients with different RCC types were almost the same; however, patients significantly differed in terms of sex and pathological T and M stages. Furthermore, 55.2% of cases had an unknown N stage. The American Joint Committee on Cancer prognostic groups (from stage I to IV) (11) and grades (from grade 1 to 4) were assigned to each case. Both stage (**Figure 1B**) and grade (**Figure 1C**) significantly increased in the following order: round < lobular < irregular. Consistent with these findings, disease-free survival (DFS) and overall survival (OS) were significantly worse in the same order [round < lobular < irregular type (**Figures 1D, E**)]. Surprisingly, no patients with round-type RCC experienced disease recurrence.

**Table 1:** Patient background comparison for each morphological classification of ccRCC in TCIA data sets.

	Radiological morphology (type)			
	Round	Lobular	Irregular	p value
Number of patients (%)	51 (31.3)	50 (30.7)	62 (38.0)	
Median age	59	59	59	
Sex				
Female	17 (33.3)	9 (18)	26 (41.9)	0.0251
Male	34 (66.7)	41 (82)	36 (58.1)	
Pathological T stage (%)				
1a	35 (68.6)	17 (34.0)	3 (4.8)	< 0.0001
1b	11 (21.6)	10 (20.0)	10 (16.1)	
2	1 (2.0)	6 (12.0)	11 (17.7)	
3	4 (7.8)	16 (32.0)	38 (61.3)	
4	0 (0.0)	1 (2.0)	0 (0.0)	
N stage				
0	21 (41.2)	20 (40.0)	28 (45.2)	0.6314
1	2 (3.9)	0 (0.0)	1 (1.6)	
X	28 (54.9)	30 (60.0)	33 (53.2)	
M stage				
0	50 (98.0)	45 (90.0)	47 (75.8)	0.0016
1	1 (2.0)	5 (10.0)	15 (24.2)	

The relationship between DFS or OS and unfavorable clinicopathological factors on univariate and multivariate analyses are shown in **Table 2**. Stage IV and grades 3 and 4 ccRCC with irregular RM were associated with significantly shorter DFS and OS in the univariate analyses. In addition, multivariate analysis revealed that the irregular subtype was a significant independent prognostic factor for DFS (*p* = 0.0307), whereas stage IV RCC was a significant independent prognostic factor for both DFS and OS (both *p* < 0.0001) (**Table 2**).

**Table 2:** Univariate and multivariate analyses of different parameters for disease-free survival (DFS) and overall survival (OS).

DFS		Univariate analysis			
		OR	95% CI		*p* value
			Lower limit	Upper limit	
RM	(Ir vs. Ro, Lo)	4.434	2.2721	9.1507	< 0.0001
Stage	(IV vs. I - III)	17.2602	8.5563	34.8481	< 0.0001
Grade	(3, 4 vs. 1, 2)	4.6238	1.9644	13.5496	0.0002
DFS		Multivariate analysis			
		OR	95% CI		*p* value
			Lower limit	Upper limit	
RM	(Ir vs. Ro, Lo)	2.22	1.0756	4.8056	0.0307
Stage	(IV vs. I - III)	10.1394	4.8338	21.5415	< 0.0001
Grade	(3, 4 vs. 1, 2)	2.3995	0.9737	7.2384	0.0577
OS		Univariate analysis			
		OR	95% CI		*p* value
			Lower limit	Upper limit	
RM	(Ir vs. Ro, Lo)	4.9288	2.066	13.5952	0.0002
Stage	(IV vs. I - III)	10.6593	4.7654	24.7444	< 0.0001
Grade	(3, 4 vs. 1, 2)	6.9946	2.0608	43.6497	0.0006
OS		Multivariate analysis			
		OR	95% CI		*p* value
			Lower limit	Upper limit	
RM	(Ir vs. Ro, Lo)	1.9068	0.71	5.7321	0.2057
Stage	(IV vs. I - III)	6.1157	2.5307	15.6792	< 0.0001
Grade	(3, 4 vs. 1, 2)	3.4355	0.9463	22.117	0.0622

### Genomic profile comparison between each RM

**Figure 2:**
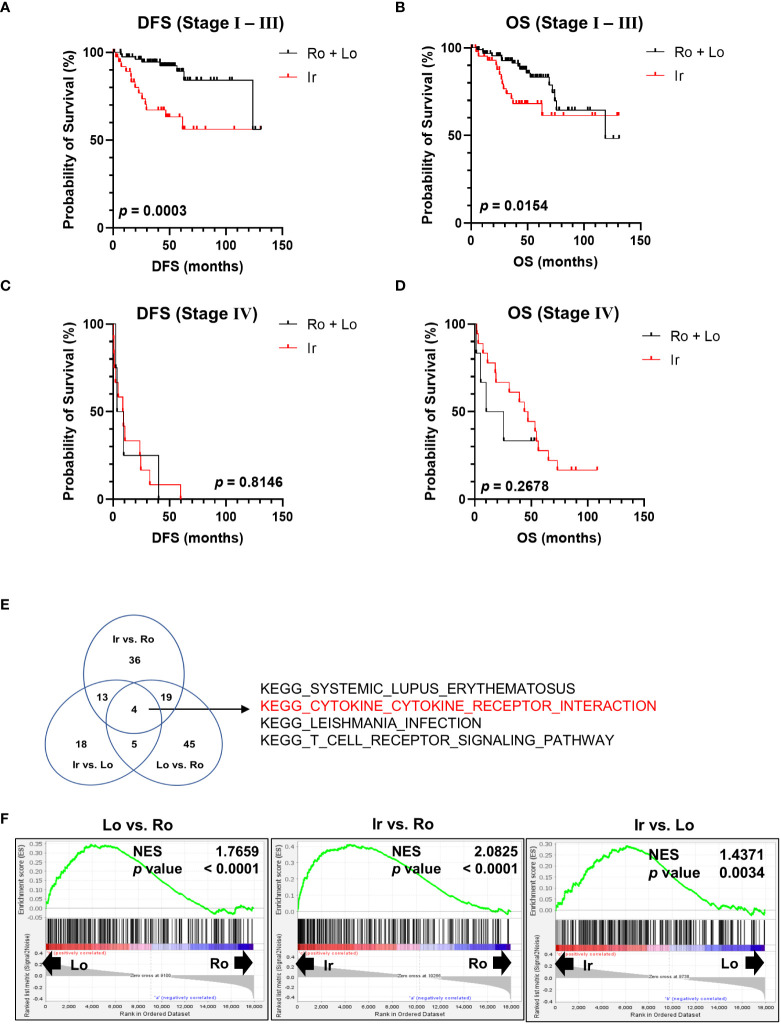
Genomic profile comparison between each RM. **(A, B)** Comparison of disease-free survival (DFS) and overall survival (OS) between the irregular and round plus lobular type RM in patients with stage I, II, and III ccRCC. **(C, D)** Comparison of DFS and OS between irregular type and round plus lobular type RM in patients with stage IV ccRCC. **(E, F)** Comparison of all three RM types (irregular vs. round type, irregular vs. lobular type, and lobular vs. round type) between stages I, II, and III using Gene Set Enrichment Analysis and Kyoto Encyclopedia of Genes and Genomes (KEGG) gene sets. **(E)** Venn diagram showing the number of gene sets that were significantly positively enriched in the left-hand type of each comparison. **(F)** Normalized enrichment score (NES) of “KEGG CYTOKINE-CYTOKINE RECEPTOR INTERACTION” in each comparison group.

Using the clinical data of 163 patients in TCGA-KIRC datasets, survival rates were calculated at each stage to further clarify the characteristics of RM. Patients with irregular RM displayed worse DFS (**Figure 2A**) and OS (**Figure 2B**) compared to patients with stages I to III lobular and round RM (**Figures 2C, D**). This trend was not observed in stage IV ccRCC.

Next, RNA-sequencing data of the patients in TCGA-KIRC datasets were subjected to Gene Set Enrichment Analysis to further investigate the molecular changes underlying the differences between RM of patients with stages I, II, and III ccRCC. RM types were compared using KEGG gene sets as follows: irregular vs. round type, irregular vs. lobular type, and lobular vs. round type. The Venn diagram shown in **Figure 2E** displays the number of gene sets significantly positively enriched in the left-hand type of each comparison. Four gene sets were common between all comparisons (**Figure 2E**). Of note, the “KEGG CYTOKINE-CYTOKINE RECEPTOR INTERACTION” gene set contained multiple inflammatory marker genes such as those encoding cytokines and chemokines that are associated with cancer prognosis (12, 13). **Figure 2F** shows a normalized enrichment score of “KEGG CYTOKINE-CYTOKINE RECEPTOR INTERACTION” in each comparison group.

### Genomic profile comparison between irregular type and the others

**Figure 3:**
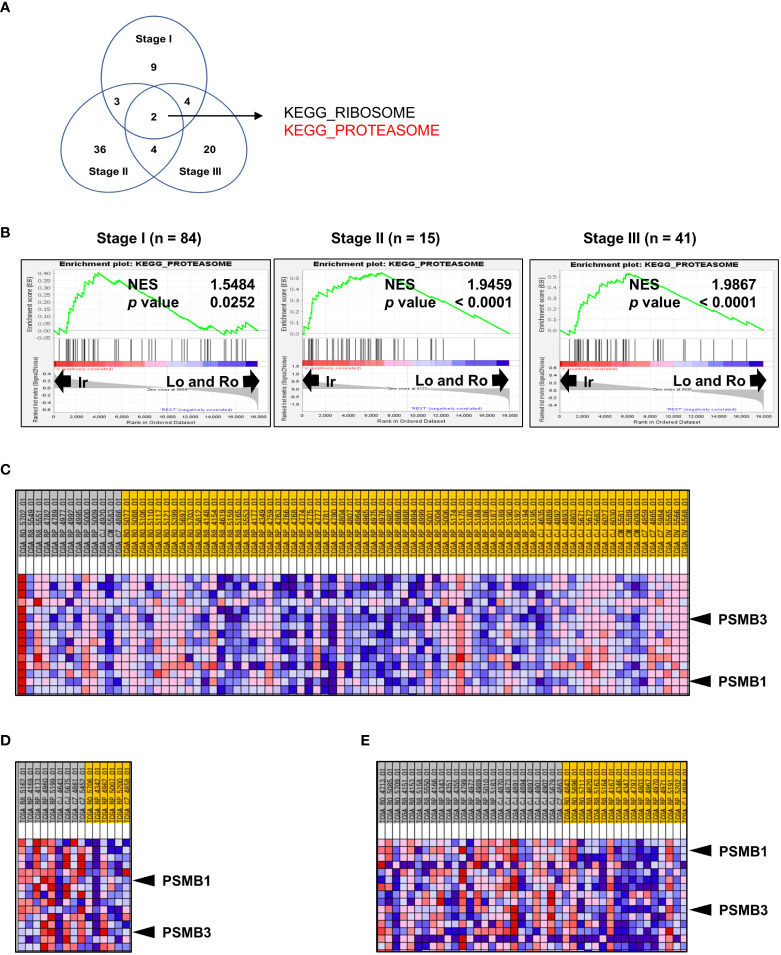
Genomic profile comparison between irregular type and the others. **(A, B)** Comparison of the irregular type with the other RM types for ccRCC stages I, II, and III using GSEA. **(A)** Venn diagram showing the number of KEGG gene sets that were significantly positively enriched in the irregular type. **(B)** Normalized enrichment score (NES) of “KEGG PROTEASOME” at each stage. **(C–E)** Blue-Pink O’ Gram of the gene set “KEGG PROTEASOME” was limited to the top 15 genes whose expression was strongly biased toward the irregular type of stage I **(C)**, stage II **(D)**, and stage III. **(E)** Only PSMB1 and PSMB3 (two non-catalytic components of the 20S core proteasome complex) were common in all comparisons.

Since the percentage of each RM type is significantly correlated with stage and grade (**Figures 1B, C**), the results shown in **Figure 3E** do not necessarily highlight the influence of RM alone. Thus, to further investigate the molecular background of RM in the same datasets used in the previous section, we compared stages I, II, and III with irregular RM to the other types using Gene Set Enrichment Analysis. The Venn diagram in **Figure 3A** shows the number of significantly positively enriched KEGG gene sets corresponding to the irregular type. Two gene sets were common among all comparison groups (**Figure 3A**), of which one codes for the proteasome subunit proteins that are strongly implicated in cancer. **Figure 3B** shows the normalized enrichment score of “KEGG PROTEASOME” at each stage. To select particularly relevant genes among the proteasome subunits, the Blue-Pink O’ Gram of the gene set limited to the top 15 genes whose expression was strongly biased toward the irregular type was used, as shown in **Figure 3C** (stage I), **3D** (stage II), and **3E** (stage III). Of the 15 genes, only two non-catalytic components of the 20S core proteasome complex, *PSMB1*, and *PSMB3* were common among all comparison groups. The patients were divided into two groups based on the median expression levels of *PSMB1* or *PSMB3*, then the relationship between DFS and unfavorable clinicopathological factors on multivariate analyses were performed (**Supplementary Table 1**). Multivariate analysis revealed that the high expression of *PSMB3* was a significant independent prognostic factor for DFS (p = 0.0356), while no statistically significant difference was reached for high expression of *PSMB1* levels (p = 0.0547).

### Association of proteasome components with RM in ccRCC

**Figure 4:**
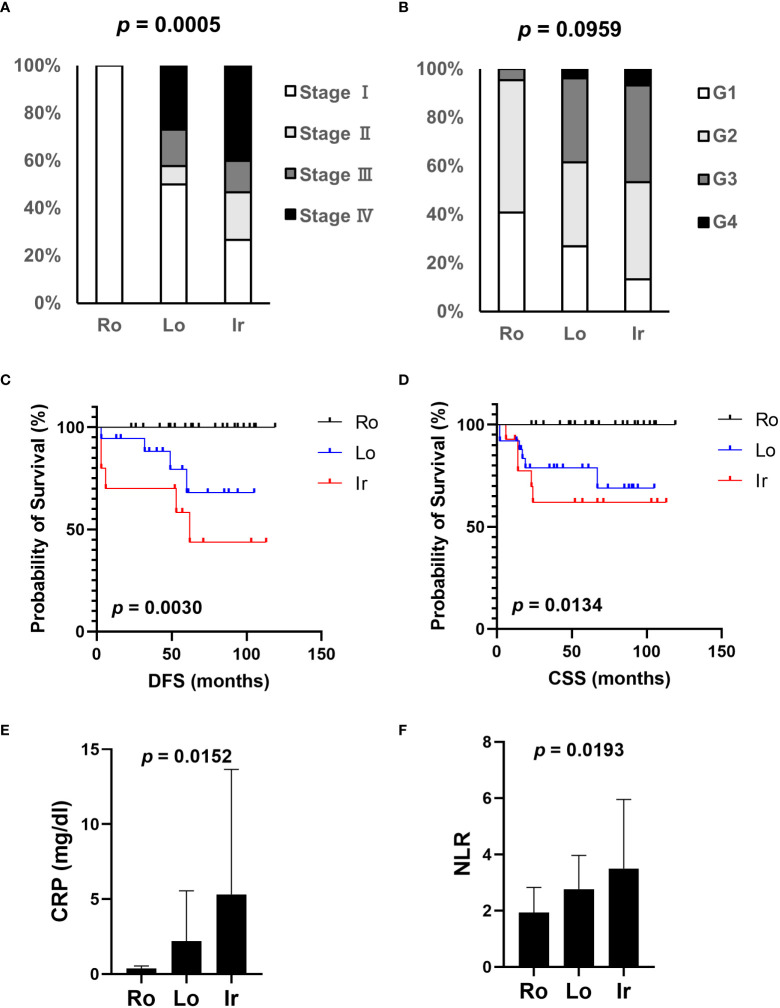
Association of clinical parameters and RM at our institution. The association between clinical parameters and RM was evaluated using data from patients with ccRCC who received radical, cytoreductive, or partial nephrectomy at our institution. **(A)** The relationship between each RM type and cancer stage. **(B)** The relationship between each RM type and cancer grade. **(C)** Comparison of disease-free survival (DFS) between each RM type. **(D)** Comparison of overall survival (OS) between each RM type. **(E, F)** Systemic inflammation-related markers, CRP level **(E)**, and NLR **(F)** were evaluated in each RM type through preoperative blood tests. Ro, round type; Lo, lobular type; Ir, irregular type; CRP, C-reactive protein; NLR, neutrophil-lymphocyte ratio.

Based on our previous results, we evaluated the association between proteasome components (PSMB1 and PSMB3) and RM using specimens obtained from 63 patients with ccRCC at our institution. Clinicopathological features of all three types of RM were compared (**Table 3**). Similar to our findings using TCIA datasets, cancer stage (**Figure 4A**) increased in the following order: round < lobular < irregular, although cancer grade was not statistically significant (**Figure 4B**). DFS and OS were significantly worse in the same order [round < lobular < irregular (**Figures 4C, D**)]. No patient with round type experienced recurrence. Based on our findings from **Figures 2E, F**, we examined the relationship between RM and the preoperative markers of the systemic inflammatory response: C-reactive protein and neutrophil-to-lymphocyte ratio (14–17). Both c-reactive protein level and neutrophil-to-lymphocyte ratio were significantly elevated in RCC in the following order: round < lobular < irregular (**Figures 4E, F**).

**Table 3:** Patient background comparison for each morphological classification of ccRCC in our institution.

	Radiological morphology (type)			
	Round	Lobular	Irregular	p value
Number of patients (%)	22 (34.9)	26 (41.3)	15 (23.8)	
Median age (range)	65 (34 - 78)	66 (45 - 86)	65 (47 - 76)	0.4739
Sex (%)				
Female	6 (27.3)	10 (38.5)	3 (20.0)	0.4333
Male	16 (72.7)	16 (61.5)	12 (80.0)	
Pathological T stage				
1a	17 (77.3)	6 (23.0)	1 (6.7)	<0.0001
1b	5 (22.7)	8 (30.8)	3 (20.0)	
2	0 (0.0)	2 (7.7)	3 (20.0)	
3	0 (0.0)	8 (30.8)	8 (53.3)	
4	0 (0.0)	2 (7.7)	0 (0.0)	
N stage				
0	22 (100.0)	23 (88.5)	13 (86.7)	0.3693
1	0 (0.0)	2 (7.7)	2 (13.3)	
X	0 (0.0)	1 (3.8)	0 (0.0)	
M stage				
0	22 (100.0)	19 (73.1)	9 (60.0)	0.0075
1	0 (0.0)	7 (26.9)	6 (40.0)	

**Figure 5:**
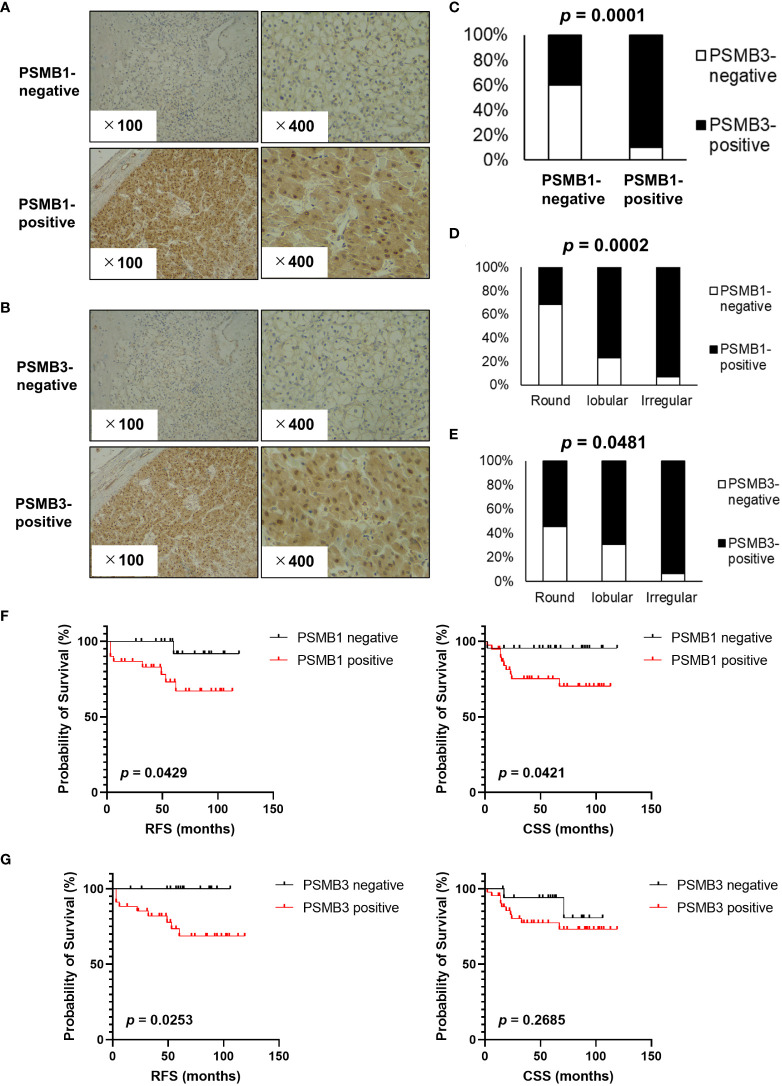
Evaluation of PSMB1 and PSMB3 expression in ccRCC. Immunohistochemistry of PSMB1 and PSMB3 was performed using specimens obtained from patients with ccRCC who received radical, cytoreductive, or partial nephrectomy at our institution. **(A, B)** Typical findings of positive and negative results are shown for PSMB1 **(A)** and PSMB3 **(B)**. **(C)** Results of chi-square test comparing the immunohistochemistry of PSMB1 and PSMB3. **(D, E)** Both PSMB1-positive **(D)** and PSMB3-positive **(E)** cases were significantly more common in the round, followed by lobular and then irregular types. **(F)** PSMB1-positive cases had significantly worse recurrence-free survival and cancer-specific survival. **(G)** PSMB3-positive cases also had significantly worse recurrence-free survival. DFS, disease-free survival.

Next, immunohistochemistry analysis of PSMB1 and PSMB3 was performed; the results are shown in **Figure 5A** (PSMB1) and **5B** (PSMB3). PSMB1-positive cases were significantly more common in PSMB3-positive cases (**Figure 5C**) than in PSMB3-negative cases. Of note, both PSMB1-positive and PSMB3-positive cases were significantly more common in the irregular type, followed by lobular and round type RCCs (**Figures 5D, E**). PSMB1-positive cases had significantly worse recurrence-free survival and cancer-specific survival rates than PSMB1-negative cases (**Figure 5F**). PSMB3-positive cases also had significantly worse recurrence-free survival (**Figure 5G**).

### Proteasome components and drug sensitivity in advanced ccRCC

According to our results shown in **Figure 1**, irregular type RCC accounted for 68.2% of stage IV RCC. To investigate the association between proteasome component expression and drug sensitivity in stage IV ccRCC, we used the clinical and genomic data from the JAVELIN RENAL 101 phase 3 clinical trial (10) that included previously untreated patients with advanced ccRCC. Expression of *PSMB1* and *PSMB3* was reported in 726 patients. Patients were divided into three groups based on the median expression levels of *PSMB1* and *PSMB3*: “low expression of both genes”, “high expression of both genes”, and “otherwise” groups. The number of patients treated with sunitinib was 127, 151, and 94 in “low expression of both genes”, “high expression of both genes”, and “otherwise” groups, respectively. The number of patients treated with avelumab plus axitinib was 107, 119, and 128 in “low expression of both genes”, “high expression of both genes”, and “otherwise” groups, respectively.

**Figure 6:**
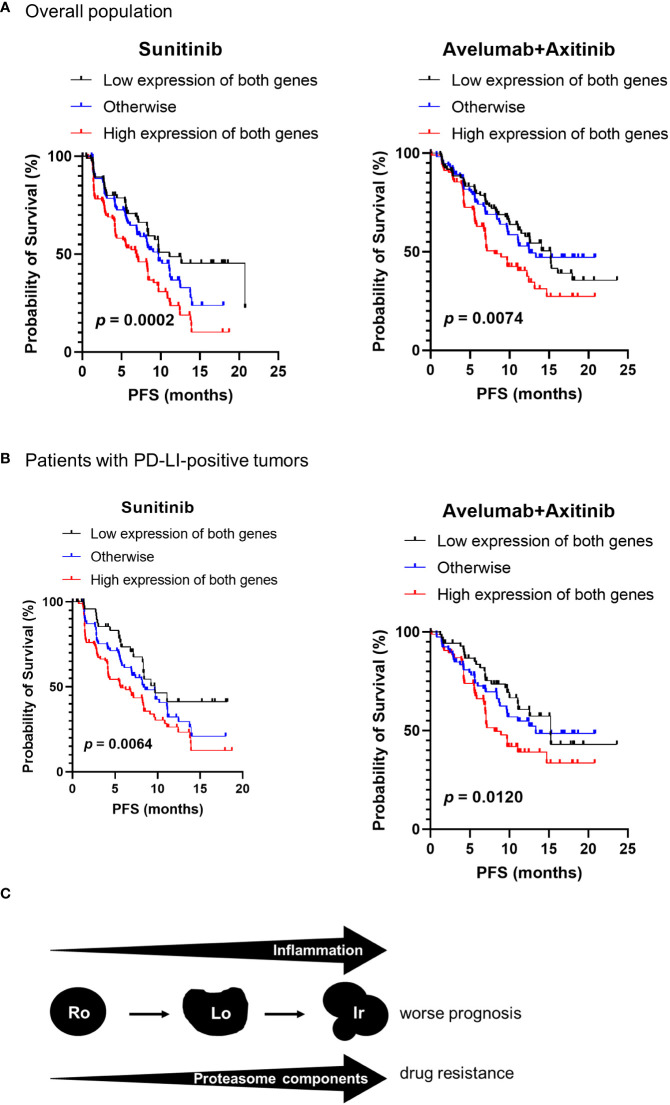
Proteasome components and drug sensitivity in advanced ccRCC. Clinical and genomic data from JAVELIN RENAL 101 phase 3 clinical trial were used. The patients were classified into three groups based on the median expression levels of *PSMB1* and *PSMB3* as “low expression of both genes”, “high expression of both genes”, and “otherwise” groups. **(A)** The “high expression of both genes” group showed significantly worse progression-free survival (PFS) in patients treated with sunitinib and with avelumab plus axitinib. **(B)** Similar results were obtained on comparing PFS in each group when restricted to patients with PDL1-positive tumors. **(C)** Schematic image of our results in this study.

The “High expression of both genes” group showed significantly worse progression-free survival in patients (**Figure 6A**). The results were similar when only patients with PDL1-positive tumors were included (**Figure 6B**).

## Discussion

Our group previously documented that RM of clinical T1 RCC can predict pathological upstaging (8). This study built on our previous report and documented the implications of RM in patients with all stages of ccRCC. Overall, increased RM complexity was associated with higher grade and stage. It would be consistent with the impression many urologists get from the imaging features of RCC. However, we found that the irregular ccRCC type was a more significant independent predictor of worse DFS than round and lobular types. Moreover, the irregular type showed significantly increased expression of the proteasome subunit proteins PSMB1 and PSMB3. Our results also indicate that the expression of such proteasome components was not necessarily specific for high grade or high stage tumors, and suggest that increased proteasome expression plays a major role in determining the relationship between RM complexity and adverse outcomes in ccRCC.

Our results also showed that inflammation-related genes and biomarkers increased as the complexity of RM increased from round to irregular. In addition, previous reports indicated that inflammatory biomarker levels increased as the ccRCC stage or grade increased (15, 16), further highlighting the importance of determining RM to predict the malignant potential of ccRCC. A schematic of our findings is shown in **Figure 6C**.

Ubiquitin–proteasome pathway likely functions as a master regulator of the overall inflammatory response (18). However, the relationship between increased RM complexity (from round to irregular type) and increased expression of proteasome and its components remains poorly understood. Intratumor heterogeneity (ITH) may further our understanding of the relationship between RM complexity and prognosis. ITH, also known as clonal heterogeneity, refers to the genetic diversity of subclones within a single tumor (19). ITH in ccRCC was evident at the RNA expression level, wherein expression signatures of good or poor prognosis were detected in different regions of the same tumor (20). In addition, although ccRCC can display a homogeneous appearance at the macroscopic level, microscopic and immunohistochemical ITH may exist (21). Previous studies have highlighted that high ITH is associated with a worse prognosis in many solid tumors, including ccRCC (22). Thus, we hypothesize that cancer cell clones with relatively uniform growth patterns possibly exhibit round or lobular type, reflecting low ITH, whereas a mixture of clones with different growth patterns possibly exhibits irregular type, reflecting high ITH. However, no study has yet investigated the direct relationship between proteasome expression and ITH, and thus, further analysis is required.

We also documented that increased expression of the proteasome components PSMB1 and PSMB3 confer resistance to VEGFR-TKI therapy in stage IV ccRCC, and previous studies have reported that overexpression of PSMB1/2/3/4/6/8/9/10 was associated with worse ccRCC prognosis (23). Consistent with our findings, a previous study reported that *PSMD1* or *PSMD3* knockdown resulted in a greater reduction of growth in TKI-resistant multiple myeloma cells than in their TKI-sensitive counterparts (24). In 2003, the US Food and Drug Administration approved the use of the proteasome inhibitor bortezomib for treating patients with multiple myeloma (25). Currently, three proteasome inhibitors, bortezomib, carfilzomib, and ixazomib, have received regulatory approval and are routinely used (26). Unfortunately, a phase II study of carfilzomib reported negative safety and efficacy findings that do not favor its use in treating RCC (27). However, a proof-of-concept study investigating *in vivo* antitumoral activity of carfilzomib using patient-derived xenografts has indicated that patient-individualized *in vitro* drug screening and preclinical validation are feasible (28). Thus, investigating RM characteristics may clarify the underlying response mechanism to proteasome inhibitors, allowing the selection of an appropriate patient population in future studies.

This study has certain limitations that need to be addressed. First, our classification of RM is qualitative; therefore, to analyze the relationship between RCC and RM more accurately, a quantitative tool may be necessary to assess the continuous change in morphological complexity from round to irregular. Additionally, recent emerging techniques of radiomics and texture analysis provide more objective and quantitative details for medical images, including CT scans (29). Such analysis would be useful in future studies.

RM analysis on pre-treatment CT scans is an effective method to predict the prognosis. Increased RM complexity may indirectly predict drug sensitivity *via* increased expression of PSMB1 and PSMB3 in patients with ccRCC. Specific targeting of the ubiquitin-proteasome system might be a novel treatment strategy for ccRCC with increased RM complexity.

## Data availability statement

The datasets presented in this study can be found in online repositories. The names of the repository/repositories and accession number(s) can be found in the article/**Supplementary Material**.

## Ethics statement

The studies involving human participants were reviewed and approved by The ethics committee in Hiroshima University. The patients/participants provided their written informed consent to participate in this study.

## Author contributions

KK and KI designed the study. KK, KI, TB, YK, RT, KT, TF, SM, HK, KG, and KH provided clinical information and performed pathological examinations. KK and KI analyzed the RNA-seq data. KK, KI, JT, and YS interpreted the results. KK, KI, TH, and NH edited the manuscript. All authors agree to be accountable for the content of the work. All authors contributed to the article and approved the submitted version.

## Supplementary material

Supplementary Figure 1 Immunohistochemistry of PSMB1 and PSMB3 was performed using specimens obtained from patients with ccRCC who received radical, cytoreductive, or partial nephrectomy at our institution. **(A)** Typical findings of negative results are shown for PSMB1 and PSMB3, with normal tubular cells stained and no cancer cells stained in the upper part of A. The magnified view of the tubules are shown in the lower part of A. **(B)** Typical findings of positive results are shown for PSMB1 and PSMB3, with normal tubular cells stained and cancer cells also stained in the upper part of B. The magnified views of the tubules are shown in the lower part of B. The dotted lines show the normal tissue boundaries, including the tubules. N, normal tissue including tubules; T, tumor.
