# Supplementary figures and images for: Complexity in radiological morphology predicts worse prognosis and is associated with an increase in proteasome component levels in clear cell renal cell carcinoma
Source: Front Oncol. 2022 Dec 8;12:1039383. doi: 10.3389/fonc.2022.1039383 (PMC9773190; doi:10.3389/fonc.2022.1039383)

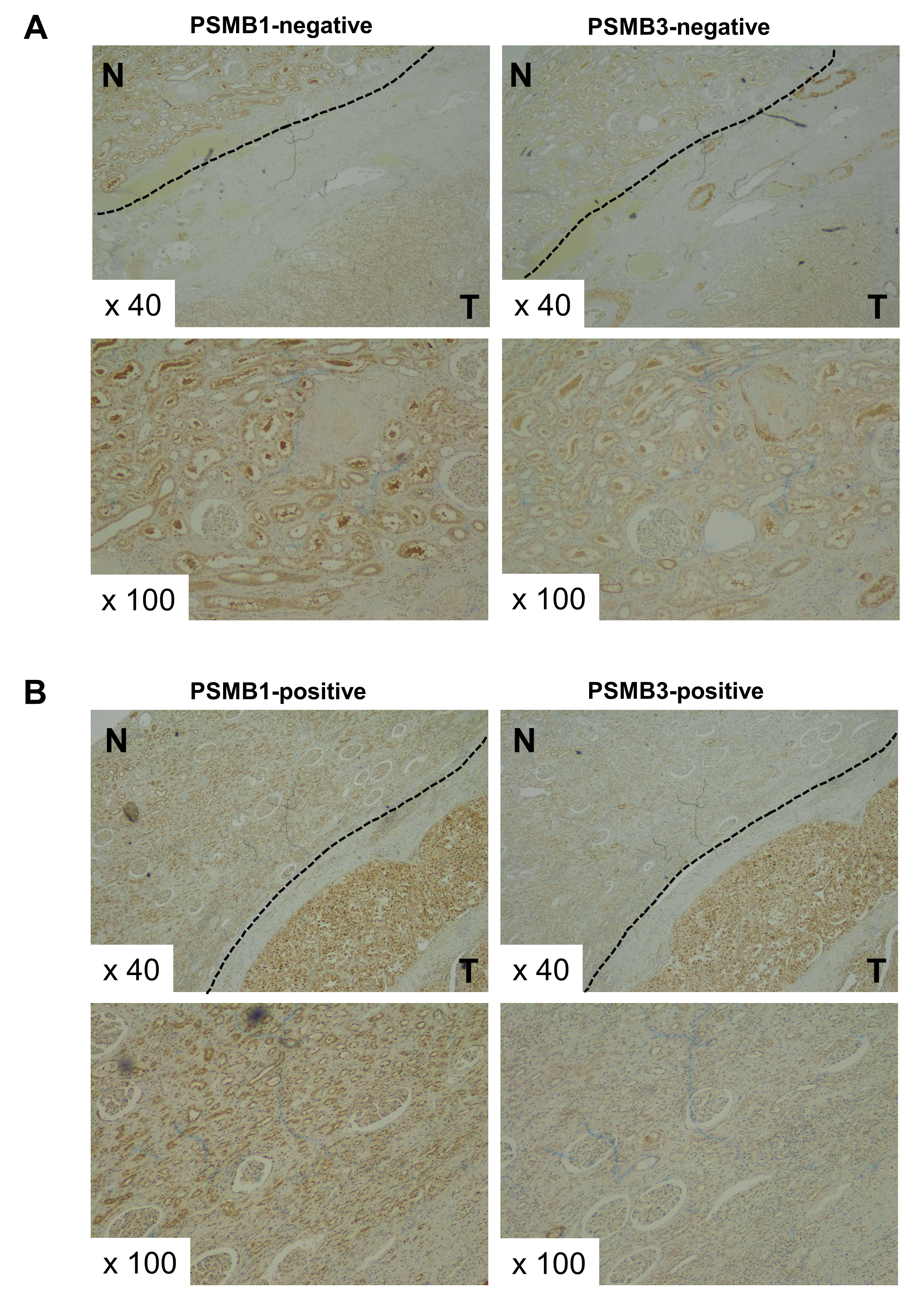

Supplement: Supplementary Figure 1 — Immunohistochemistry of PSMB1 and PSMB3 was performed using specimens obtained from patients with ccRCC who received radical, cytoreductive, or partial nephrectomy at our institution. (A) Typical findings of negative results are shown for PSMB1 and PSMB3, with normal tubular cells stained and no cancer cells stained in the upper part of A. The magnified view of the tubules are shown in the lower part of A. (B) Typical findings of positive results are shown for PSMB1 and PSMB3, with normal tubular cells stained and cancer cells also stained in the upper part of B. The magnified views of the tubules are shown in the lower part of B. The dotted lines show the normal tissue boundaries, including the tubules. N, normal tissue including tubules; T, tumor. [file Image_1.tif]
