# Supplementary material for: Complexity in radiological morphology predicts worse prognosis and is associated with an increase in proteasome component levels in clear cell renal cell carcinoma
Source: Front Oncol. 2022 Dec 8;12:1039383. doi: 10.3389/fonc.2022.1039383 (PMC9773190; doi:10.3389/fonc.2022.1039383)
Supplement: Supplementary file 2 [file Table_1.docx]

**Supplementary table 1**

Multivariate analyses of different parameters including expression of *PSMB1* or *PSMB3* for disease-free survival (DFS)

(*PSMB1*)

| DFS |  | Multivariate analysis | | | |
| --- | --- | --- | --- | --- | --- |
|  |  | OR | 95% CI | | *p* value |
|  |  |  | Lower limit | Upper limit |  |
| *PSMB1* | (high vs. low) | 1.4274 | 0.9920 | 2.0678 | 0.0547 |
| Stage | (Ⅳ vs. Ⅰ - Ⅲ) | 7.6975 | 5.2301 | 11.2387 | < 0.0001 |
| Grade | (3, 4 vs. 1, 2) | 2.6805 | 1.7833 | 4.1419 | < 0.0001 |

(*PSMB3*)

| DFS |  | Multivariate analysis | | | |
| --- | --- | --- | --- | --- | --- |
|  |  | OR | 95% CI | | *p* value |
|  |  |  | Lower limit | Upper limit |  |
| *PSMB3* | (high vs. low) | 1.5077 | 1.0276 | 2.2435 | 0.0356 |
| Stage | (Ⅳ vs. Ⅰ - Ⅲ) | 7.3773 | 4.9969 | 10.8151 | < 0.0001 |
| Grade | (3, 4 vs. 1, 2) | 2.6248 | 1.7432 | 4.0618 | < 0.0001 |
